# Elevated Mortality among Birds in Chernobyl as Judged from Skewed Age and Sex Ratios

**DOI:** 10.1371/journal.pone.0035223

**Published:** 2012-04-11

**Authors:** Anders Pape Møller, Andrea Bonisoli-Alquati, Geir Rudolfsen, Timothy A. Mousseau

**Affiliations:** 1 Laboratoire d’Ecologie, Systématique et Evolution, CNRS UMR 8079, Université Paris-Sud, Bâtiment 362, Orsay, France; 2 Department of Biological Sciences, University of South Carolina, Columbia, South Carolina, United States of America; 3 Norwegian Radiation Protection Authority (NRPA), Department of Environmental Radioactivity, The Fram Center, Tromsø, Norway; University of Bern, Switzerland

## Abstract

**Background:**

Radiation has negative effects on survival of animals including humans, although the generality of this claim is poorly documented under low-dose field conditions. Because females may suffer disproportionately from the effects of radiation on survival due to differences in sex roles during reproduction, radiation-induced mortality may result in male-skewed adult sex ratios.

**Methodology/Principal Finding:**

We estimated the effects of low-dose radiation on adult survival rates in birds by determining age ratios of adults captured in mist nets during the breeding season in relation to background radiation levels around Chernobyl and in nearby uncontaminated control areas. Age ratios were skewed towards yearlings, especially in the most contaminated areas, implying that adult survival rates were reduced in contaminated areas, and that populations in such areas could only be maintained through immigration from nearby uncontaminated areas. Differential mortality in females resulted in a strongly male-skewed sex ratio in the most contaminated areas. In addition, males sang disproportionately commonly in the most contaminated areas where the sex ratio was male skewed presumably because males had difficulty finding and acquiring mates when females were rare. The results were not caused by permanent emigration by females from the most contaminated areas because none of the recaptured birds had changed breeding site, and the proportion of individuals with morphological abnormalities did not differ significantly between the sexes for areas with normal and higher levels of contamination.

**Conclusions/Significance:**

These findings are consistent with the hypothesis that the adult survival rate of female birds is particularly susceptible to the effects of low-dose radiation, resulting in male skewed sex ratios at high levels of radiation. Such skewed age ratios towards yearlings in contaminated areas are consistent with the hypothesis that an area exceeding 30,000 km^2^ in Chernobyl’s surroundings constitutes an ecological trap that causes dramatic excess mortality.

## Introduction

The effects of low-dose radiation under field conditions on mortality of animals including humans remain poorly understood. As a case in point, available estimates of the excess mortality of humans due to the nuclear catastrophe at Chernobyl range from less than 50 cases [1] to close to one million [2]. The main reason is that some scientists believe that excess human mortality (and morbidity in general) in the neighborhood of Chernobyl is mainly of psychological origin rather than caused by negative effects of radiation [3]–[5]. However, free-living animals also show significant increases in mortality. For example, Møller et al. [6] used standard capture-mark-recapture procedures in barn swallows *Hirundo rustica* to estimate adult survival rates, as already done for hundreds of bird species worldwide including several populations of barn swallows in Europe. The barn swallow is particularly suitable for such analyses because adults are extremely philopatric to their once chosen breeding site with hardly no individuals ever leaving a once chosen breeding site [7]. Adult survival rate for male barn swallows in areas of Ukraine impacted by the Chernobyl accident was 0.327, while it was 0.431 in uncontaminated control areas, equaling a reduction by 24% [6]. For females the corresponding estimates were 0.233 and 0.542, respectively, or a reduction by 57%. This sex by radiation effect was highly significant implying that females suffered disproportionately from high levels of radiation. Because barn swallows are philopatric after their first breeding event, we can exclude the possibility that the sex by radiation effect was due to permanent emigration [6]. A possible mechanism is the greater investment of antioxidants by females into eggs [8], causing depletion of antioxidants in contaminated areas [9], where radiation results in elevated levels of oxidative stress [10] with likely consequences for reproduction. Similarly strong negative effects of radiation have been reported for another bird species from Chernobyl [11]. Because birds are unknown to worry about the effects of radiation on their health (the cause invoked to explain excess mortality in humans in contaminated areas in Ukraine, Belarus and Russia [3]–[5]), this hypothesis is an unlikely explanation for the morbidity reported in birds.

If females suffer from differential mortality due to radiation, as suggested by the data on barn swallows [6], we should generally expect male skewed sex ratios in contaminated, but not in uncontaminated control areas among all species of birds. This prediction has not been tested. A second consequence of differential female mortality in contaminated areas is an excess of unmated males in the predominantly socially monogamous birds. Because unmated males attempt to attract a mate by producing song [12], we should expect that singing males are more common in areas with higher levels of background radiation. Again, this simple prediction has so far not been tested. Birds constitute a superb model system for such tests because there is already available information on adult survival rate and other demographic variables for most common species. Here we provide such a test.

The specific objectives of this study were to test (1) whether adult survival rates were lower in more contaminated areas; (2) whether sex ratios in adults were skewed towards males; and (3) whether the fraction of birds that were singing increased in areas with high levels of radiation. In addition, we test the assumption that differential mortality is not caused by permanent emigration of females from contaminated areas by comparing recapture rates and dispersal distances in areas with normal and higher levels of contamination. In addition, morphological abnormalities are very common in contaminated areas around Chernobyl, but uncommon elsewhere in nature [13, A. P. Møller et al. unpublished data]. If females emigrated permanently from the more contaminated areas, there should be a deficit of females with abnormalities in the more contaminated areas compared to area with normal background radiation levels.

## Methods

### Study Sites and Breeding Bird Survey

APM (wearing a radiation protection suit in the most contaminated areas, although not using the hood to prevent obstructing observing and hearing the birds) conducted standard point counts during May–June 2006–2009, with each count lasting five minutes during which all birds seen or heard were recorded, including information on whether the individual was singing [14]–[17]. The survey between 0700 and 1900 local time was conducted within the Chernobyl Exclusion Zone (or in areas adjacent on the southern and western borders) with a permit from the Ukrainian authorities and in areas in southern Belarus around Gomel (Fig. 1 in [15]). A total of 898 survey points were located at ca. 100 m intervals within forested areas. Survey results are highly consistent among years as shown by analyses of abundance and species richness [18].

We surveyed birds at the beginning of June when most individuals reach their annual maximum of singing activity, making surveys of breeding birds highly reliable [19]. The degree of consistency was high for both species richness and abundance when testing the reliability of our counts by letting two persons independently perform counts for a subset of our surveys [15]. Most birds surveyed using point counts are located within a distance of 100 m from the observer [14], [15], [19].

**Figure 1:**
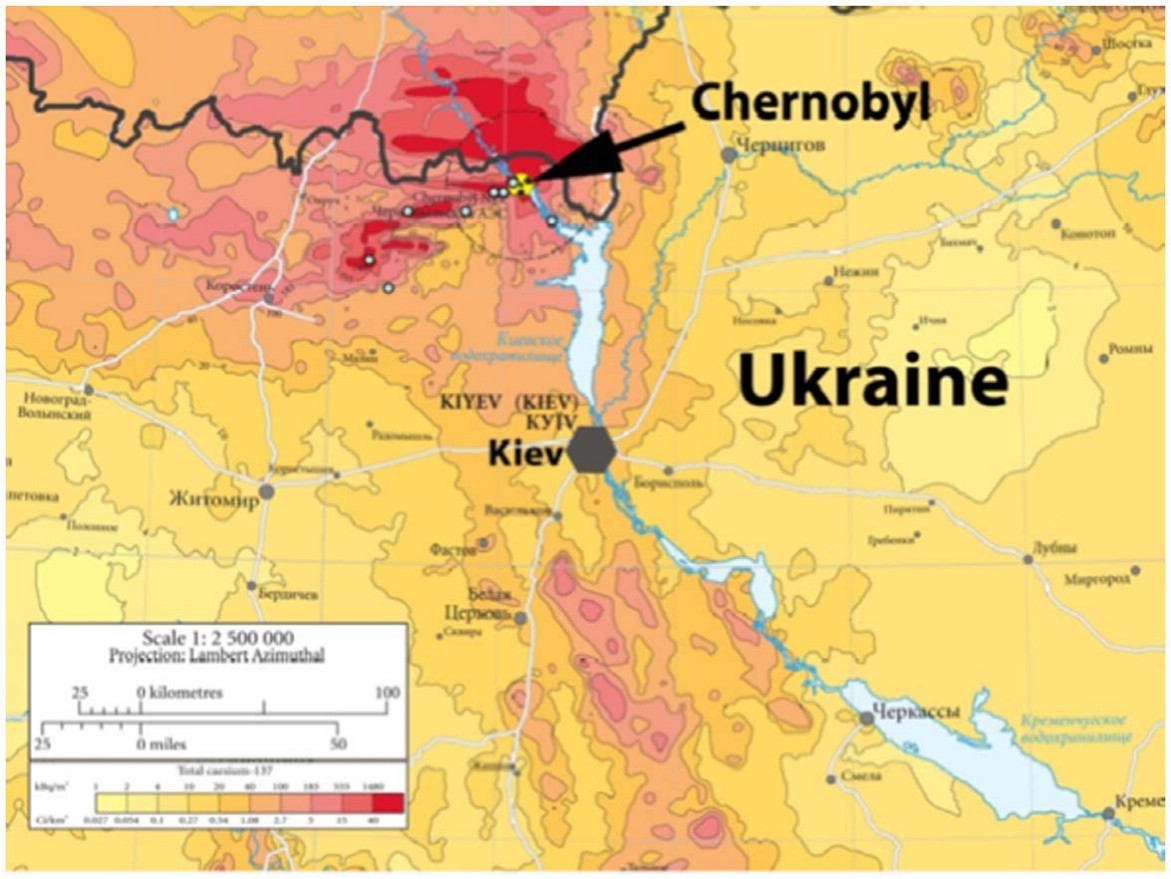
Background radiation in the Chernobyl region and location of study sites. Adapted from European Union [**31**].

### Trapping birds for aging and sexing

We captured 1080 birds (546 birds in 2010 and 534 in 2011) using 35 × 12 m mist nets placed in woodland habitat that have previously been shown to exhibit severe reductions in species richness and density of birds and other vertebrates and invertebrates [16]–[18], [20]. The radiation levels at these trapping sites ranged from 0.02 to 138 µSv/h, where values in the range 0.02–0.05 µSv/h constitute normal background radiation levels in Northern Ukraine in areas uncontaminated by radionuclides from Chernobyl. These captures were made in eight different areas around Chernobyl, Ukraine during 25 May – 5 June 2010 and 29 May – 11 June 2011. Each area was used for capture of birds on two consecutive days each of the two years (Fig. 1).

All birds were sexed and aged according to criteria in Svensson [21]. Snow [22], and more recently Ricklefs [23], have shown that such age ratios (the proportion of all birds being yearlings) provides reliable information on adult survival rate when the age of first reproduction is one year as is the case for the generally small bird species investigated here.

In addition to the captures listed above we captured breeding barn swallows at farms varying in level of radiation within and outside the Chernobyl Exclusion Zone, with a total of 2466 captures during 1991, 1995 and 2000–2009 (see [6] for details). All adults were assumed to be yearlings when first captured because barn swallows almost always recruit to the breeding population when one year old [6], [7]. These data on barn swallows were pooled with the data on the other species because we tested if age and sex ratios of birds in general were correlated with background radiation.

All captured birds were ringed with a numbered aluminum ring to allow individual identification and to allow estimates of recapture probability in subsequent years.

### Measuring Background Radiation Levels

Breeding birds generally remain within their territory during the breeding season [24]. We measured radiation levels in the field at the survey points and the exact trapping locations of of each individual bird, and the radiation level at the actual site of capture will provide information about the radiation level experienced by that individual during breeding due to the sedentary habits of breeding birds. We measured α, β, and γ radiation at ground level at each capture location and each survey point after having conducted the survey (thus making the survey blindly with respect to radiation level) using a hand-held dosimeter (Model: Inspector, SE International, Inc., Summertown, TN, USA). We measured levels several (2–3) times at each site one measurement right after each other and averaged the measurements. Our data were validated with correlation against data from the governmental measurements published by Shestopalov [25], estimated as the mid-point of the ranges published, with analyses showing a high degree of consistency between methods [16]. Radiation levels vary greatly at a local scale due to heterogeneity in deposition of radioactive material after the Chernobyl accident [25]. Our measurements at the survey points ranged from 0.01 to 380 µSv/h. Because we had small samples at each radiation level, we also conservatively conducted the analyses using aggregated age and sex ratio data on a logarithmic scale ranging from less than 0.1 µSv/h, 0.1–0.2 µSv/h, 0.2–0.4 µSv/h, 0.4–0.8 µSv/h, etc. up to above 327.68 µSv/h. Thus we pooled all individuals captured at these intervals of radiation. Background radiation levels are strongly positively correlated with internal dose levels for individual birds in Chernobyl [26].

### Adult Survival Rates

We recorded annual adult survival rate from Europe using Cramp and Perrins [24] as a source for the species recorded at Chernobyl. While there is no information on survival rates from the study area, we assumed that survival estimates from elsewhere in Europe would also be representative for uncontaminated areas in the Chernobyl region. This assumption is supported by a highly significant repeatability of adult survival rate based on 299 survival estimates obtained for 94 species of European birds, implying significantly more variation among than within species (*F*  = 14.52, d.f.  =  93, 204, *r* ^2^  =  0.87, *P* < 0.0001). If multiple estimates were provided, we extracted the information from the UK because that estimate generally was based on the largest sample sizes.

In a hypothetical stable population, dead adults are replaced by a similar number of yearlings, so recruitment equals mortality. Therefore, we can estimate the expected proportion of yearlings from the mean adult survival rate (s) for the different species with the proportion of yearlings being (1-s) [22], [23]. This expected value can be compared with the observed relative frequency of yearlings in a given species at different levels of radiation. The assumption about populations being stable is supported by the mean population trend of 124 species of birds across the European continent equaling -0.0050 (SE  =  0.0026), which is very close to zero (data obtained from the European Bird Census Council at http://www.ebcc.info/index.php?ID=457). The frequency distribution of these population trends is close to normal with most species having stable population trends with values around zero.

### Recaptures of Birds

A total of 546 adult birds were individually ringed in 2010, and the ringing site of all recaptured birds in 2011 was determined to assess local breeding dispersal distance. The distance between the two most distant study sites exceeded 100 km, which represented the maximum possible breeding dispersal distance.

### Recording Morphological Abnormalities

Morphological abnormalities such as partial albinism and deformed beaks, eyes, toes and feathers are rare in nature, but occur at a high frequency around Chernobyl [13]. We carefully inspected all captured birds for signs of abnormalities as previously described in detail for barn swallows [13].

### Statistical Analyses

Radiation level was log_10_-transformed to normalize the variable.

In the analyses of age ratios we developed the following logistic regression model:
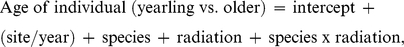
where the nested term in brackets is a random effect.

We subsequently tested if the observed mortality rate based on published estimates of adult survival rate estimates from the literature differed significantly from the proportion of yearlings in control sites with normal levels of background radiation and in contaminated sites with high levels of radiation. The underlying assumptions are that the mortality rate should equal the proportion of yearlings in the control sites, but not in the contaminated sites, and that permanent emigration by females has not increased differentially in the most contaminated areas. An absence of a significant difference in control sites would also provide a test of the assumption that trapping probability was unbiased by age effects. If there were an age-bias in trapping probability, the difference between observed and expected proportions of yearlings would be significantly different from zero. For these analyses we used paired t-tests based on square-root arcsine-transformed proportions of yearlings. We explicitly tested the second assumption about emigration by determining if dispersal among our study sites differed between areas with normal and higher levels of contamination, respectively. In addition, we tested if the proportion of individuals with abnormalities differed between the sexes, and if this difference was larger in areas with higher levels of contamination than in areas with normal levels, as would be expected if females differentially emigrated from the most contaminated areas.

Second, we developed a logistic regression model to assess the relationship between sex ratio and radiation, species and the radiation by species interaction as predictor variables, assuming a binomial distribution, as implemented in the statistical software JMP [27]. The model had the following structure:
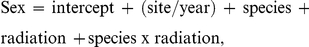
where the nested term in brackets is a random effect. We also developed a more conservative statistical model based on grouped radiation levels on a logarithmic scale. In this model the square-root arcsine-transformed sex ratio was the response variable and the log_10_-transformed radiation level the predictor variable. In these models we assumed that the sex bias in capture probability remained constant across radiation levels. This untested assumption is based on the fact that birds or other organisms, perhaps with the exception of a few radio-resistant fungi, are unable to sense radiation.

We tested if presence of morphological abnormalities differed between sexes and level of contamination using the following logistic regression model:
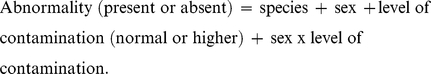



In the analyses of singing birds we developed the following logistic regression model:
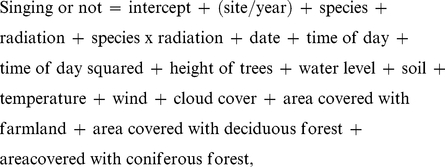
where the nested term in brackets is a random effect. The environmental variables are defined and described in detail elsewhere [16]. Model selection was based on Akaike’s Information Criterion (AIC) starting with a full model, using the criterion of delta AIC < 2.00 for a variable being of minor importance [28].

Finally, we tested if the proportion of singing birds was related to the sex ratio by estimating the two variables for the classes of background radiation of less than 0.1 µSv/h, 0.1–0.2 µSv/h, 0.2–0.4 µSv/h, 0.4–0.8 µSv/h, etc. up to above 327.68 µSv/h. Thus the entire data set was reduced to 17 pairs of observations on sex ratio and proportion of singing birds, respectively.

## Results

### Age Ratios and Radiation

**Figure 2:**
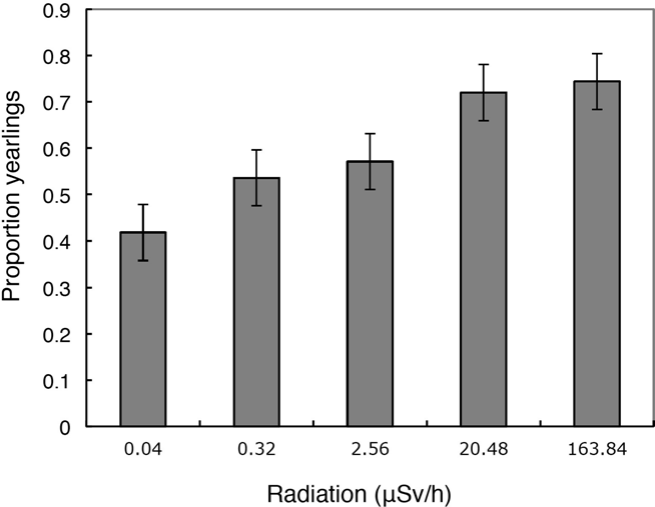
Observed age ratio (proportion of all birds that were yearlings) of birds captured in mist nets in relation to level of background radiation (µSv/h) during 2010–2011. Error bars represent standard errors.

The overall percentage of yearlings was 60.6% of 760 individuals among the species with data for both normal (< 0.05 µSv/h) and higher radiation levels (> 0.05 µSv/h)(Table 1). Whether individuals were yearlings or older was positively related to level of background radiation (Fig. 2; Wald χ^2^  =  27.79, d.f.  =  1, *P* < 0.0001, slope (SE)  =  0.432 (0.082)) in a model that also included species (Wald χ^2^  =  63.35, d.f.  =  33, *P*  =  0.0012) and year as factors (Wald χ^2^  =  6.90, d.f.  =  1, *P*  =  0.0086). There was no significant additional effect of sex (Wald χ^2^  =  2.89, d.f.  =  1, *P*  =  0.09), or sex by radiation interaction (Wald χ^2^  =  0.31, d.f.  =  1, *P*  =  0.58).

**Table 1:** Age ratio (% yearlings), sample size and difference between adult survival rate and adult survival rate estimate based on age ratio (100 - % yearlings) for areas with normal (< 0.05 µSv/h) and higher levels of background radiation ((≥ 0.05 µSv/h) during 2010–2011.

Species	Age ratio normal radiation	N	Age ratio higher radiation	N	Adult survival rate	Difference in survival rate at normal radiation	Difference in survival rate at higher radiation
*Carduelis chloris*	0.00	2	100.00	1	43.0	–57.00	43.00
*Emberiza citrinella*	80.00	5	66.67	6	53.0	33.00	19.67
*Erithacus rubecula*	36.36	22	74.00	50	38.0	–25.64	12.00
*Ficedula hypoleuca*	100.00	1	100.00	4	49.9	49.90	49.90
*Fringilla coelebs*	25.00	32	40.30	67	64.0	–11.00	4.30
*Hirundo rustica*	65.93	226	37.50	36	37.0	2.93	–25.50
*Luscinia luscinia*	0.00	1	37.50	24	48.5	–51.50	–14.00
*Motacilla alba*	0.00	1	70.58	17	52.0	–48.00	22.58
*Parus caeruleus*	50.00	8	80.00	5	41.6	–8.40	21.60
*Parus major*	30.00	20	65.85	41	48.6	–21.40	14.45
*Parus palustris*	60.00	5	100.00	5	55.0	15.00	55.00
*Phoenicurus ochruros*	50.00	4	66.67	15	55.3	5.30	21.97
*Sylvia atricapilla*	52.63	19	68.42	19	46.0	–1.37	14.42
*Troglodytes troglodytes*	50.00	2	100.00	1	37.0	–13.00	37.00
*Turdus merula*	47.06	17	69.84	63	56.0	3.06	25.84
*Turdus philomelos*	46.15	19	90.91	22	46.0	–7.85	36.91
Mean	43.32		73.02		48.18	–8.50	21.20
SE	7.01		5.42		1.92	7.20	5.34
*T*						–1.18	3.97
*P*						0.26	0.003

Northern Ukraine has low levels of background radiation around 0.01–0.05 µSv/h in areas that are unaffected by contamination from Chernobyl [25]. The null expectation for difference in survival rate is zero, while the predicted difference is expected to be zero for normal background radiation, but a significantly lower difference survival rate at higher levels of background radiation.

Mean adult survival rate for the species recorded at Chernobyl is 0.504 (SE  =  0.014), *N*  =  57 species, according to Cramp & Perrins [24]. In stable populations where recruitment equals mortality, as estimated above, yearlings will constitute 0.496 of all adults in the population (1 – 0.504). While this estimate of 0.496 is close to the observed frequency of yearlings at normal radiation levels below 0.05 µSv/h, the observed relative frequency of yearlings equal to or above 0.05 µSv/h was much larger than predicted (Fig. 2).

The percentage of yearlings among the 16 species with samples for both normal (< 0.05 µSv/h) and higher radiation levels (≥ 0.05 µSv/h) was 43.32% at radiation levels < 0.05 µSv/h, while it was 73.02% at radiation levels ≥ 0.05 µSv/h. Adult survival rates estimated from these age ratios did not differ significantly from published adult survival rates at radiation levels < 0.05 µSv/h, but did differ significantly from adult survival rates estimated from age ratios at radiation levels ≥ 0.05 µSv/h (Table 1). In addition, the observed minus the expected survival rates from areas with < 0.05 µSv/h and ≥ 0.05 µSv/h differed from each other (paired t-test, *t*  =  3.85, d.f.  =  15, *P*  =  0.0016). Restricting these analyses to species with a sample size greater than ten did not change any of these conclusions.

Among the 64 recaptures in 2011 of the 546 birds ringed in 2010 all were recaptured in the same site as where they were ringed. Thus, there were no cases of breeding dispersal among sites.

Presence or absence of morphological abnormality differed significantly among species (Wald χ^2^  =  103.30, d.f.  =  58, *P*  =  0.0002) and it depended on whether areas had normal or higher levels of contamination (Wald χ^2^  =  24.90, d.f.  =  1, *P* < 0.0001, slope (SE)  =  0.996 (0.200)). In contrast, there was no significant effect of sex (Wald χ^2^  =  0.10, d.f.  =  1, *P*  =  0.75) or sex by contamination interaction (Wald χ^2^  =  0.04, d.f.  =  1, *P*  =  0.84). Thus there was no evidence that the frequency of females with abnormalities was lower in areas with higher levels of contamination, as compared to areas with normal levels of background radiation.

### Sex Ratios and Radiation

**Figure 3:**
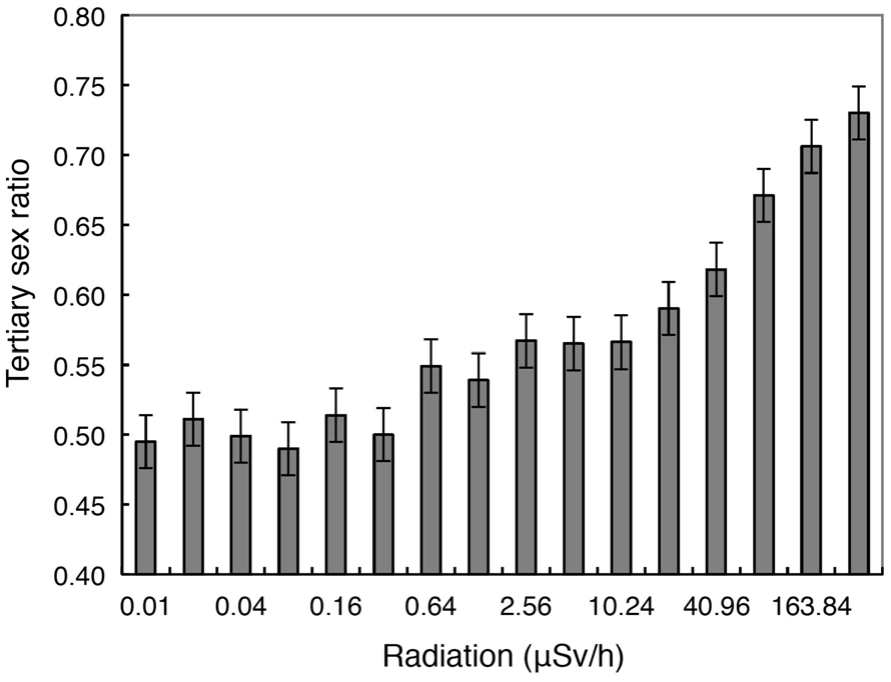
Observed adult tertiary sex ratio (proportion of all adult birds that were males) of birds captured in mist nets in relation to level of background radiation (µSv/h) during 2010–2011. Error bars represent standard errors.

The overall tertiary sex ratio (the proportion of all individuals being males) of mist netted birds was 0.539 (N  =  3546). There was a significant positive relationship between radiation and the probability of being male (Wald χ^2^  =  10.96, d.f.  =  1, *P*  =  0.0009, slope (SE)  =  0.119 (0.036)). Species did not account for additional significant variation (Wald χ^2^  =  46.99, d.f.  =  58, *P*  =  0.85). Likewise, the partial effect of year was not significant (Wald χ^2^  =  20.14, d.f.  =  1, *P*  =  0.06). If we grouped observations into logarithmic intervals of radiation (see Methods), the tertiary sex ratio increased from 0.495 in the least contaminated control areas to 0.730 in the most contaminated areas (Fig. 3; *F*  =  77.37, d.f.  =  1,14, *r* ^2^  =  0.85, *P* < 0.0001, slope (SE)  =  0.050 (0.006)).

### Singing Birds and Radiation

**Figure 4:**
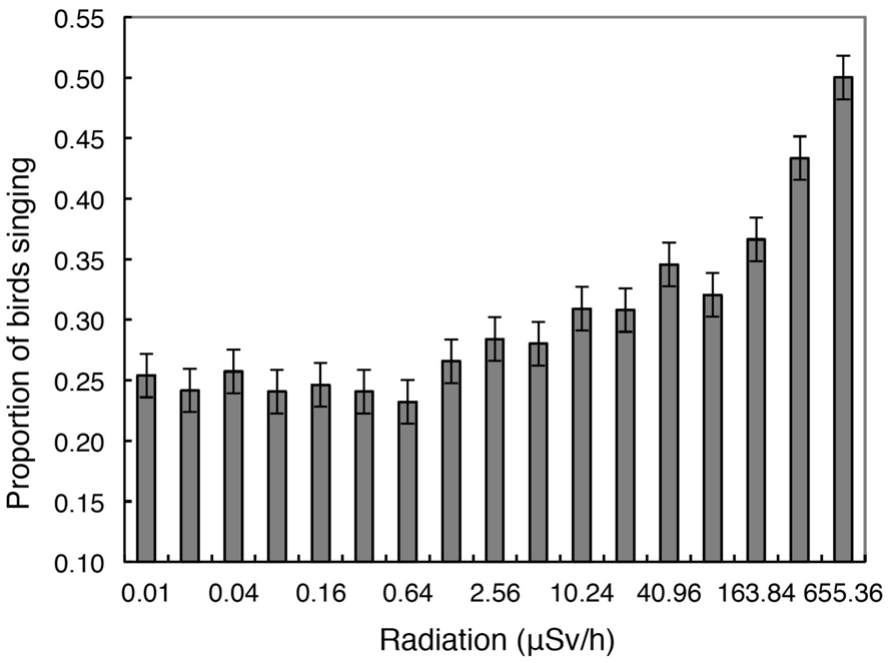
Observed proportion of individual birds singing of all birds recorded during point count breeding bird surveys in relation to level of background radiation (µSv/h) during 2006–2009. Error bars represent standard errors.

A total of 26.6% of 6296 individuals were recorded as singing during the point counts. The fraction of individuals singing increased with increasing radiation level, ranging from 25% in uncontaminated areas to 50% in the most contaminated areas when we grouped observations into classes of radiation (Fig. 4; *F*  =  43.47, d.f.  =  1,15, *r* ^2^  =  0.74, *P* < 0.0001, slope (SE)  =  0.045 (0.007)). A number of potentially confounding variables could account for the positive relationship between the fraction of singing birds and the level of background radiation. Hence, it was important to assess if the relationship remained even after controlling statistically for these potentially confounding variables. A model that controlled for these confounding variables (see Methods) showed a strong effect of radiation on singing (likelihood ratio χ^2^  =  114.89, *P* < 0.0001, estimate (SE)  =  0.465 (0.043)). This implies that the relationship between the fraction of singing birds and the level of background radiation was not caused by these potentially confounding variables.

The relationship between the fraction of singing birds and the tertiary sex ratio estimated from the ringing data was strongly positive when using aggregated data into logarithmic intervals of radiation (see Methods) (*F*  =  88.37, d.f.  =  1,14, *r* ^2^  =  0.86, *P* < 0.0001, slope (SE)  =  0.715 (0.076)). This suggests that as the tertiary sex ratio increased above the level of parity, an increasing fraction of birds were recorded as singing during breeding bird surveys.

## Discussion

The main finding of this study was that yearlings constituted an increasing fraction of birds with increasing radiation levels, these findings being consistent with the hypothesis that adult mortality increased with radioactive contamination, while there was no evidence of these effects being caused by emigration. Furthermore, the tertiary sex ratio (here defined as the proportion of males among adults) was skewed towards an excess of males across 48 different bird species. This finding is in accordance with lower adult survival rates in females compared to males previously reported for barn swallows in contaminated areas around Chernobyl [6]. We could exclude the possibility of differential emigration by females from contaminated sites causing this effect because all recaptured birds were found in their original site of capture, and because the frequency of females with morphological abnormalities did not differ between sites with normal and sites with higher levels of contamination. Finally, singing birds constituted a larger fraction of all surveyed breeding birds at high background radiation levels compared to control areas. This observation is consistent with a male-biased sex ratio causing males to sing for a longer time in order to attract a mate. These three principal findings are all novel, and they suggest that there are significant life history costs arising from low dose radiation.

Barn swallows from Chernobyl have reduced adult survival rates compared to conspecifics from uncontaminated control areas in Ukraine, and these differences are not caused by differential emigration by females because we have not recorded a single case of a barn swallow moving from one breeding site to another between years [6]. Here we extended this result by testing for a reduction in adult survival rate among 16 species of birds. Using age ratios of captured birds, we showed a reduction in adult survival rate by 40% in contaminated areas, but no difference between observed and expected adult survival rates based on Western European estimates when analyzing age ratios from uncontaminated areas in Ukraine. The estimated reduction in adult survival rate of barn swallows was 43%, similar to the value of 40% that we have previously reported based on capture-mark-recapture analyses [6]. We could exclude the possibility that this difference was caused by permanent emigration as shown by an absence of breeding dispersal among sites, and by no sex difference in the frequency of abnormalities between sites with normal and with higher levels of contamination. Although the conservation consequences of the Chernobyl disaster, and more recently the Fukushima disaster, are poorly known, they include reductions in the distribution and abundance of rare species and increases in the frequency of selective deaths due to mutations [29]. We can now start making estimates of the conservation implications of radiation exposure using adult survival rates as estimated from age ratios from the field for a large number of bird species. First, we know that populations in contaminated areas are maintained through immigration from source populations, because local reproduction and survival are insufficient for maintenance of stable populations [30]. Second, we have shown reduced adult survival rates [6; this study] and reproductive rates [6], [11] in contaminated areas. Age ratios for birds at normal and high background radiation levels differed considerably from an average of 43% at normal levels to an average of 73% at high levels. This observed difference in age ratios between control and contaminated areas implies an increase in the frequency of juveniles from 43% to 73%, or an increase by 30% (73% - 43%). Given that the area contaminated by more than 0.05 µSv/h exceeds 30,000 km^2^ (Fig. 1; [31]), and given that the typical population density of breeding birds exceeds 100 pairs per km^2^ [24], this implies an annual excess mortality of 30,000 km^2^ × 100 pairs/km^2^ × 0.3  =  1.8 million birds. These findings also imply that the magnitude of this ecological sink is likely to exceed that of any other sinks described in the literature (review in [32]). Therefore, the findings reported here also have significant conservation consequences caused by the Chernobyl disaster. Finally, skewed sex ratios may increase the risk of local extinction for demographic reasons [33], [34].

Female barn swallows suffer differentially from the mortality costs of radiation, with male adult survival being reduced by 24%, while female survival is reduced by 57% in contaminated areas compared to controls [6]. Thus differential mortality of females in contaminated areas, as shown for the barn swallow [6], causes male-skewed tertiary sex ratios. If such sex differences in survival rate were common, we should expect sex ratios among adults to be skewed towards males. Here we have reported such a skew across a large number of species, using captures of adult birds during the main breeding season, with the sex ratio increasing from parity at normal background radiation to more than 73% males at the highest radiation levels. We can exclude the possibility that these estimates were biased because we used mist netting to obtain samples of birds for sexing and aging. Mist netting is a reliable tool for research due to low observer bias, high detection probability of species that are often missed using other survey methods and ease of standardized sampling [35]. The hypothesis of differential mortality in female birds due to greater expenditure of antioxidants [8] as a cause of male-skewed sex ratios is also consistent with the observation that bird species in which females invest the most in production of eggs through maternal allocation of antioxidants to eggs are the species that experience the strongest reduction in abundance of breeding birds due to radiation [18].

Birds sing to attract mates and repel potential competitors [12]. We have shown here that the fraction of singing birds of all birds surveyed during breeding bird point counts increased with level of radiation. We assumed that all singing birds were males, and this is likely to generally be the case (review in [36]). All singing individuals that were sexed using binoculars in the present study only revealed singing males. These findings have important implications for breeding bird surveys. Based on the present study we can conclude that previous estimates of species richness and abundance of birds reported by Møller and Mousseau [16]–[18], [20] for Chernobyl are likely to be over-estimates for areas with high levels of contamination. These relative estimates of density were based on the assumption of an even sex ratio, while in fact singing, unmated males were more frequent at high background radiation levels. Thus the negative effects of background radiation on abundance and species richness of breeding birds are likely to be stronger than previously thought. We can also conclude that the high frequency of singing birds in contaminated areas occurred despite the lower density of competitors in such areas, emphasizing the importance of song for mate attraction [12].

In conclusion, we have shown significant reductions in adult survival rates for a range of bird species in areas contaminated with low-dose radiation as reflected by skewed age ratios. Because adult female survival rate is differentially affected by radiation, we expected male-skewed tertiary sex ratios in contaminated areas, as we have documented. Independently, male birds sang disproportionately often in the most contaminated areas, as expected if there was a deficit of females, and if males sing in order to attract mates. There was no evidence consistent with the alternative hypothesis that females emigrated differentially from the most contaminated sites. These results suggest significant mortality costs of low-dose radiation with severe consequences for breeding populations of birds in vast areas of contamination in Ukraine, Russia and Belarus.

## References

[1] Chernobyl Forum (2005). Chernobyl: The True Scale of the Accident..

[2] Yablokov AV, Nesterenko VB, Nesterenko AV (2009). Chernobyl: Consequences of the Catastrophe for People and the Environment.. Annals of the New York Academy of Sciences.

[3] Chernobyl Forum Expert Group ‘Environment’ (2006). Environmental consequences of the Chernobyl accident and their remediation: twenty years of experience/report of the Chernobyl Forum Expert Group ‘Environment’..

[4] Bromey EJ, Havenaar JM, Guey LT (2011). A 25 year retrospective review of the psychological consequences of the Chernobyl accident.. Clin Oncol.

[5] Rahu M (2003). Health effects of the Chernobyl accident: Fears, rumours and the truth.. Eur J Cancer.

[6] Møller AP, Mousseau TA, Milinevsky G, Peklo A, Pysanets E, Szép T (2005). Condition, reproduction and survival of barn swallows from Chernobyl.. J Anim Ecol.

[7] Møller AP (1994). Sexual selection and the barn swallow..

[8] Blount JD, Houston DC, Møller AP (2000). Why egg yolk is yellow.. Trends Ecol Evol.

[9] Møller AP, Surai PF, Mousseau TA (2005). Antioxidants, radiation and mutation in barn swallows from Chernobyl..

[10] Bonisoli-Alquati A, Mousseau TA, Møller AP, Caprioli M, Saino N (2010). Increased oxidative stress in barn swallows from the Chernobyl region.. Comp Biochem Physiol A.

[11] Møller AP, Karadas F, Mousseau TA (2008). Antioxidants in eggs of great tits *Parus major* from Chernobyl and hatching success.. J Comp Physiol B.

[12] Catchpole CK, Slater PJB (2008). Bird song..

[13] Møller AP, Mousseau TA, de Lope F, Saino N (2007). Elevated frequency of abnormalities in barn swallows from Chernobyl.. Biol Lett.

[14] Bibby CJ, Hill DA, Burgess ND, Mustoe S (2005). Bird census techniques..

[15] Voříšek P, Klvanova A, Wotton S, Gregory RD (2010). A best practice guide for wild bird monitoring schemes..

[16] Møller AP, Mousseau TA (2007). Species richness and abundance of birds in relation to radiation at Chernobyl.. Biol Lett.

[17] Møller AP, Mousseau TA (2009). Reduced abundance of raptors in radioactively contaminated areas near Chernobyl.. J Orn.

[18] Møller AP, Mousseau, TA (2007). Determinants of interspecific variation in population declines of birds from exposure to radiation at Chernobyl.. J Appl Ecol.

[19] Møller AP (1983). Methods for monitoring bird populations in the Nordic countries..

[20] Møller AP, Mousseau TA (2011). Efficiency of bio-indicators for low-level radiation under field conditions. Ecol.. Indicators.

[21] Svensson L (2006). Identification guide to European passerines..

[22] Snow DW (1956). The annual mortality of the blue tit in different parts of its range.. Brit Birds.

[23] Ricklefs RE (1997). Comparative demography of New World populations of thrushes (*Turdus* spp.).. Ecol Monogr.

[24] Cramp S, Perrins CM (1988). The birds of the Western Palearctic..

[25] Shestopalov VM (1996). Atlas of Chernobyl exclusion zone..

[26] Gaschak SP, Maklyuk YuA, Maksymenko AN, Maksymenko VM, Martynenko VI, Mousseau TA (2008). Radioactive contamination and abnormalities in small birds in the Chernobyl zone from 2003–2005..

[27] SAS Institute Inc (2000). JMP..

[28] Burnham KP, Anderson DR (2002). Model selection and multimodel inference..

[29] Møller AP, Mousseau TA (2011). Conservation consequences of Chernobyl and other nuclear accidents.. Biol Cons.

[30] Møller AP, Hobson KA, Mousseau TA, Peklo AM (2006). Chernobyl as a population sink for barn swallows: Tracking dispersal using stable isotope profiles.. Ecol Appl.

[31] European Union (1998). Atlas of Caesium deposition on Europe after the Chernobyl accident.. Bruxelles: EU Publication EUR.

[32] Robertson BA, Hutto RL (2006). A framework for understanding ecological traps and an evaluation of existing evidence.. Ecology.

[33] Møller AP, Legendre S (2001). Allee effect, sexual selection and demographic stochasicity.. Oikos.

[34] Rankin DJ, Kokko H (2007). Do males matter? The role of males in population dynamics.. Oikos.

[35] Dunn EH, Ralph CJ (2004). Use of mist nets as a tool for bird population monitoring.. Stud Avian Biol.

[36] Garamszegi LZ, Pavlova D, Eens M, Møller AP (2007). The evolution of song in female birds in Europe.. Behav Ecol.

